# Bio-synthesized TiO_2_ nanoparticles and the aqueous binder-based anode derived thereof for lithium-ion cells

**DOI:** 10.1186/s11671-024-04010-y

**Published:** 2024-04-17

**Authors:** Akhilash Mohanan Pillai, Sumol V. Gopinadh, Peddinti V. R. L. Phanendra, Patteth S. Salini, Bibin John, Sujatha SarojiniAmma, Mercy Thelakkattu Devassy

**Affiliations:** 1 Energy Systems Development Division, Energy Systems Group, PCM Entity, Vikram Sarabhai Space Centre, Thiruvananthapuram, Kerala 695022 India; 2 University of Kerala, Thiruvananthapuram, 695034 India; 3 Energy Systems Group, PCM Entity, Vikram Sarabhai Space Centre, Thiruvananthapuram, Kerala 695022 India

**Keywords:** Lithium-ion batteries, Green-synthesis, TiO_2_-NPs, Water-based binder, Electrochemical performance

## Abstract

Titanium dioxide nanoparticles (TiO_2_-NPs) are a promising anode material for Lithium-ion batteries (LIBs) due to their good rate capability, low cost, non-toxicity, excellent structural stability, extended cycle life, and low volumetric change (∼4%) during the Li^+^ insertion/de-insertion process. In the present paper, anatase TiO_2_-NPs with an average particle size of ~ 12 nm were synthesized via a green synthesis route using Beta vulgaris (Beetroot) extract, and the synthesized TiO_2_-NPs were evaluated as anode material in LIBs. Furthermore, we employed an aqueous binder (1:1 mixture of carboxy methyl cellulose and styrene butadiene) for electrode processing, making the process cost-effective and environmentally friendly. The results revealed that the Li/TiO_2_ half-cells delivered an initial discharge capacity of 209.7 mAh g^−1^ and exhibited superior rate capability (149 mAh g^−1^ at 20 C) and cycling performances. Even at the 5C rate, the material retained a capacity of 82.2% at the end of 100 cycles. The synthesis route of TiO_2_-NPs and the aqueous binder-based electrode processing described in the present work are facile, green, and low-cost and are thus practically beneficial for producing low-cost and high-performance anodes for advanced LIBs.

## Introduction

Lithium-ion batteries (LIBs) conquered the burgeoning global electric vehicle (EV) market due to their high power and energy densities and extended cycle life [1–7]. A typical LIB is composed of a cathode, anode, electrolyte, and separator. Current commercial LIBs employ LiCoO_2_ (LCO), LiMn_2_O_4_ (LMO), LiFePO_4_ (LFP), LiNi_1/3_Mn_1/3_Co_1/3_O_2_ (NMC 111), or LiNi_0.8_Mn_0.1_Co_0.1_O_2_ (NMC 811) as cathode materials, and graphite or carbonaceous materials as anode materials [8–14]. Among the various components, anode materials play a pivotal role in determining the electrochemical performance of LIBs. The widely used graphitic carbon anode suffers from several thorny issues, which hinder their commercial applications in high-power LIBs. These problems include expansion and shrinkage of graphite during the lithium-ion insertion and de-insertion process, which causes graphite particles to crack, leading to increased self-discharge or safety issues [15]. Thus researchers investigated many alternative anode-active materials, which would circumvent these limitations. As an alternative to graphitic carbon, several materials like CuO, NiO, SnO_2_, Co_3_O_4_, fiberlike-Fe_2_O_3_, Li_4_Ti_5_O_12_, Si, anatase-TiO_2_, rutile-TiO_2_, etc. have been explored [16, 17]. Among these, active materials such as Si, NiO, SnO_2_, CuO, and Fe_2_O_3_ suffer from high volume expansion and structural deformation, which leads to capacity fading. In contrast, metal oxides such as Li_4_Ti_5_O_12_, CeO_2_, and TiO_2_ exhibit relatively smaller volume expansion during the charging/discharging process [18–20].

Among the various metal oxide anode materials, TiO_2_ is considered as a promising anode candidate for LIBs, owing to its non-toxicity, low cost, high capacity, excellent structural stability, extended cycle life, and relatively small volume change (∼4%) during Li^+^ insertion/de-insertion process [21, 22]. Moreover, another intriguing aspect of TiO_2_ is that it has a greater operating potential than graphite anode (> 1.5 V vs Li^+^/Li), which could effectively inhibit the formation of lithium dendrites and lithium plating [23, 24]. TiO_2_ exists in various polymorphic forms such as brookite (space group: Pbca), anatase (space group: I41/amd), rutile (space group: P42/mnm), ramdellite (space group: Pbnm), and bronze TiO_2_-B (space group: C2/m) [25, 26]. During the insertion-deinsertion reaction, the anatase framework can accommodate 0.5 Li per formula unit with excellent insertion kinetics, whereas brookite and rutile lattices can accommodate only a small percentage of lithium ions. It is known that reducing the particle size from micron to nanoscale increases the surface area of the electrode–electrolyte interface, shortens the ion and electron paths, and thus leads to improved specific capacity and rate capability. Various synthesis strategies including sol–gel, hydrothermal, evaporation-induced self-assembly (EISA), electrospinning, etc. are employed to synthesize TiO_2_ nanoparticles (TiO_2_-NPs) [26–28]. However, these processes are intricate, energy-intensive, and challenging for mass production [29, 30]. On the contrary green synthesis has been widely endorsed, as it is devoid of the toxic effects of various chemical methods. Thus, to reduce complexity and increase process efficiency, we have adopted an easy, inexpensive, and environmentally friendly bio-synthetic approach for the synthesis of TiO_2_-NPs.

In the present work, TiO_2_-NPs have been synthesized by a green-mediated approach using Beta vulgaris (Beetroot) extract. Bio-molecules present in the extract act as both reducing agents as well as capping agents and are thus responsible for the formation of TiO_2_-NPs. The present bio-mediated process minimizes complexity and makes the process cost-effective. The biosynthesized TiO_2_-NPs were well characterized and investigated its electrochemical performance as anode material for LIB application. Moreover, in this work, we employed a water-based binder (a combination of carboxymethyl cellulose (CMC) and styrene butadiene rubber (SBR)) for electrode processing, which strongly reduces the inconveniences caused by commonly employed PVDF binder which requires toxic and costly solvents like 1-methyl-2-pyrrolidinone (NMP) for processing [31]. Hence, the present manuscript highlights a completely green approach for the synthesis as well as the electrode processing of TiO_2_-NPs.

## Materials and methods

### Reagents

Titanium isopropoxide purchased from Sigma Aldrich was used as such without further purification. Beetroot (*Beta vulgaris*) was obtained from the local market in Mangad, Kollam, Kerala.

### Extract preparation

Beta vulgaris (beetroot) was thoroughly washed with distilled water before being cut into pieces. 10 g of beetroot was boiled in 100 mL of double-deionized water for 15 min to get the extract. For the synthesis of TiO_2_-NPs, the resultant extract was cooled, filtered, and placed in a refrigerator.

### Synthesis of TiO_2_-NPs

In the field of nanotechnology, the green synthesis of nanomaterials from plants or plant component extracts has received much attention. Green-mediated nanoparticles synthesis utilizes a bottom-up approach in which synthesis occurs with the help of reducing and stabilizing agents. Physical and chemical methods usually require toxic chemicals as capping agents to maintain stability, thus leading to toxicity in the environment. Currently, researchers are employing green nanoparticle synthesis as a simple, cost-effective, non-toxic, and environment-friendly technique for the synthesis of a variety of materials. The plant extracts rich in phytochemicals can act as both reducing agent and capping or stabilization agent.

In the present study, TiO_2_-NPs were synthesized as follows: 80 ml of 0.5 M titanium isopropoxide solution in isopropyl alcohol was added to 80 ml of beetroot extract (v/v = 1:1) and stirred at 50–60 °C for 3 h. The obtained precipitate was thoroughly washed, filtered, and dried and was heated in a furnace at 500 °C for 5 h to remove the organic components. The obtained TiO_2_-NPs were thoroughly characterized.

### Material characterization

X-ray diffraction (XRD, Cu Kα radiation (λ = 0.15406 nm), Bruker D8 Discover) was employed to characterize the crystalline structure of TiO_2_-NPs over 2θ range from 10° to 80°. The crystallite size was calculated by the Debye–Scherrer equation. Scanning electron microscopy (FE-SEM, GEMINI SEM 500) was used to analyze the morphology of the prepared TiO_2_-NPs. High-resolution transmission electron microscopy (HR-TEM) and selected area electron diffraction (SAED) were used to investigate the structure of the as-prepared sample. FTIR (Nicolet iS50 FTIR) spectra were recorded to examine the characteristic functional groups. LabRAM HR Evol spectrometer was used to record Raman scattering (RS) spectra. Using the Brunauer–Emmett–Teller (BET) method, the surface area and pore volume were determined.

### Electrochemical measurements

TiO_2_-NPs, acetylene black, CMC, and SBR were mixed in the weight ratio 80:10:5:5 with de-ionized water to prepare a slurry. The obtained slurry was then coated on a copper foil and then dried under vacuum at 70 °C and calendered; then the circular discs were punched out with a diameter of 12 mm. The electrode had a mass loading of about 2.3 mg cm^−2^. Standard two-electrode CR2032-type coin cells were used to evaluate the electrochemical performance. Metallic lithium acts as both the reference and counter electrode in electrochemical measurement. The half-cell was assembled in an argon-filled glove box with a Celgard 2320 membrane as the separator, and 1 M LiPF_6_ in a mixture of ethylene carbonate (EC), diethyl carbonate (DEC), and ethyl methyl carbonate (EMC) (1:1:1, by weight) as the electrolyte. Using a NEWARE battery tester, charge/discharge cycling tests were carried out at room temperature in the constant current-constant voltage (CC-CV) mode in the voltage window of 1.0–3.0 V vs Li^+^/Li. To investigate the redox characteristics, cyclic voltammetry (CV) was carried out at a scan rate of 100 µVs^−1^ in the voltage window of 0.5 to 3.5 V. CV measurements were also carried out at various scan rates (100 µVs^−1^ to 900 µVs^−1^) to evaluate the diffusion coefficient of TiO_2_-NPs based anode.

## Results and discussions

### Role of beetroot extract in the synthesis

The precise identification of the secondary metabolites found in the beetroot used in this study has already been documented in works that are already reported in the literature. Beta vulgaris contains a variety of biologically active phytochemicals, such as betalains, flavonoids, polyphenols, saponins, and inorganic nitrate. It is also a rich source of a variety of minerals, including potassium, sodium, phosphorous, calcium, magnesium, copper, iron, zinc, and manganese [32–34]. Betanin (betanidin 5-O-β-D-glucoside, the major red beet pigment) is a betalains pigment and is used as a powerful antioxidant and coloring agent in the food industry. Due to its strong antioxidant and reducing activity, which is based on its capacity to capture free radicals, betanin may function as a reducing agent [35]. Additionally, betanin can act as a natural capping agent to stabilize metal/metal oxide nanoparticles because of its capacity to coordinate metal ions. The green synthesis of Ag/TiO_2_ nanocomposites in the presence of beetroot extract has been reported [35], where Betanin was suggested to act both as a reducing and a capping agent.

### Reaction mechanism involved in the synthesis of TiO_2_-NPs

The formation of TiO_2_-NPs in the present study involves two steps: (i) hydrolysis and (ii) condensation. Titanium isopropoxide (Ti(C_3_H_7_O)_4_), is used as the Ti^4+^ ion precursor, which usually undergoes hydrolysis in the presence of H_2_O as reported in the previous studies. The mechanism for the formation of TiO_2_-NPs is shown below [36]:

Hydrolysis

1 $${\text{Ti}}\left( {{\text{OR}}} \right)_{{4}} + {\text{ H}}_{{2}} {\text{O}} \to {\text{Ti}}\left( {{\text{OR}}} \right)_{{3}} \left( {{\text{OH}}} \right) \, + {\text{ ROH}}$$

2 $${\text{Ti}}\left( {{\text{OR}}} \right)_{{3}} \left( {{\text{OH}}} \right) \, + {\text{ H}}_{{2}} {\text{O }} \to {\text{ Ti}}\left( {{\text{OR}}} \right)_{{2}} \left( {{\text{OH}}} \right)_{{2}} + {\text{ ROH}}$$

3 $${\text{Ti}}\left( {{\text{OR}}} \right)_{{2}} \left( {{\text{OH}}} \right)_{{2}} + {\text{ H}}_{{2}} {\text{O }} \to {\text{ Ti}}\left( {{\text{OR}}} \right)\left( {{\text{OH}}} \right)_{{{3} }} + {\text{ ROH}}$$

4 $${\text{Ti}}\left( {{\text{OR}}} \right)\left( {{\text{OH}}} \right)_{{{3} }} + {\text{ H}}_{{2}} {\text{O }} \to {\text{ Ti}}\left( {{\text{OH}}} \right)_{{{4} }} + {\text{ ROH}}$$

Condensation

5 $${\text{2 TiOH}})_{{{4} }} \to \left( {{\text{OH}}} \right)_{{3}} {\text{Ti}} - {\text{O}} - {\text{Ti}}\left( {{\text{OH}}} \right)_{{3}} + {\text{ H}}_{{2}} {\text{O}}$$

6 $${\text{n }}\left( {{\text{OH}}} \right)_{{3}} {\text{Ti}} - {\text{O}} - {\text{Ti}}\left( {{\text{OH}}} \right)_{{3}} \to {\text{TiO}}_{{2}} + {\text{ nH}}_{{2}} {\text{O}}$$

where R is the isopropyl group, titanium isopropoxide condenses to produce oxopolymers, which are then converted into a TiO_2_ network in a series of stages.

**Scheme 1:**
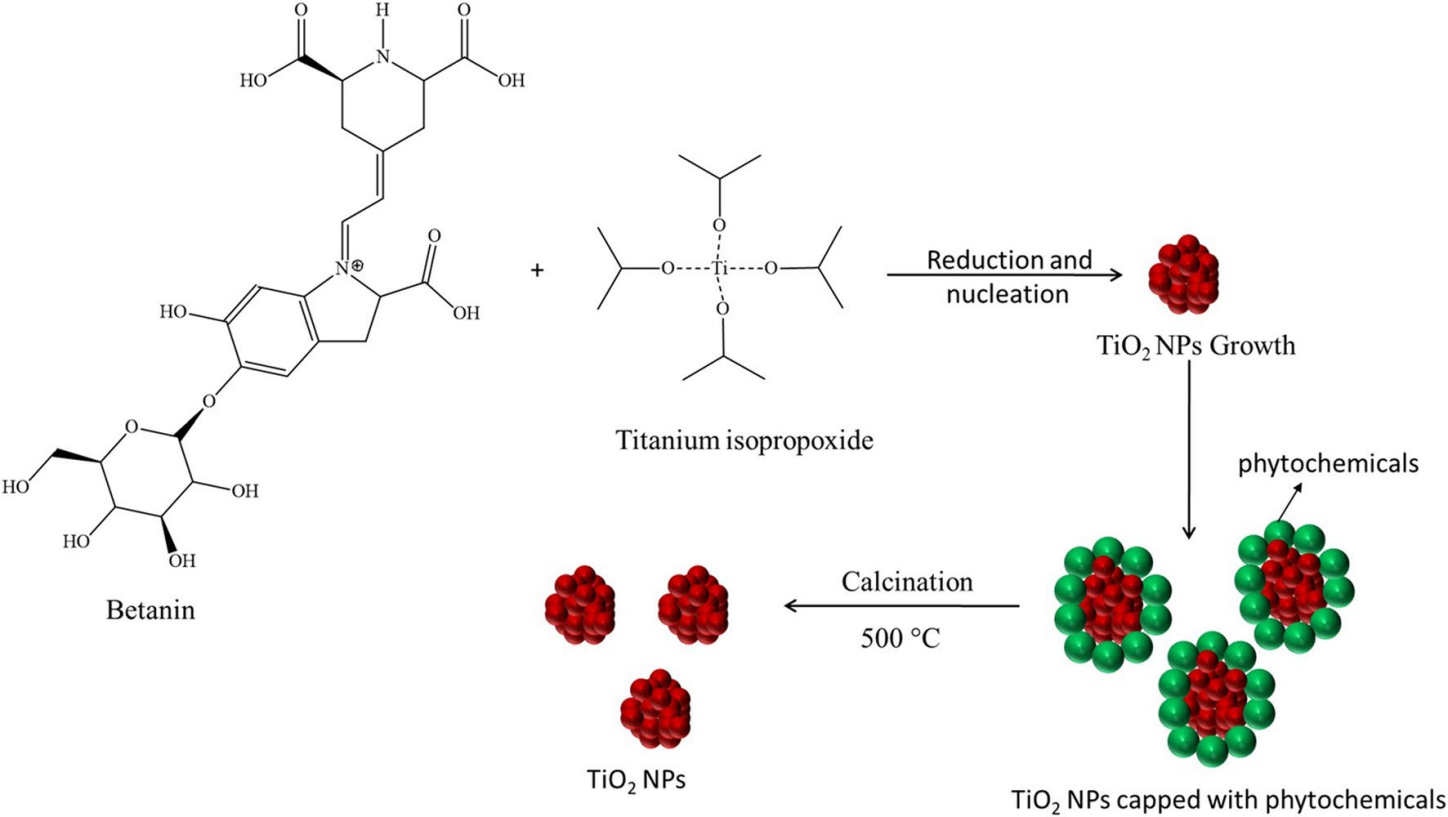
The proposed capping mechanism of the betanin on TiO_2_-NPs

The type and concentration of phytochemicals found in the extract, along with the concentration of metal alkoxide precursor, are the key factors influencing the nanoparticle growth conditions, in the current study. The chemical interaction between the surface of TiO_2_-NPs and the phenolic –OH present in the betanin may be responsible for controlling particle size and stability [36]. The proposed capping mechanism of betanin on the TiO_2_-NPs is shown in Scheme 1.

### Characterization of TiO_2_-NPs

**Fig. 1:**
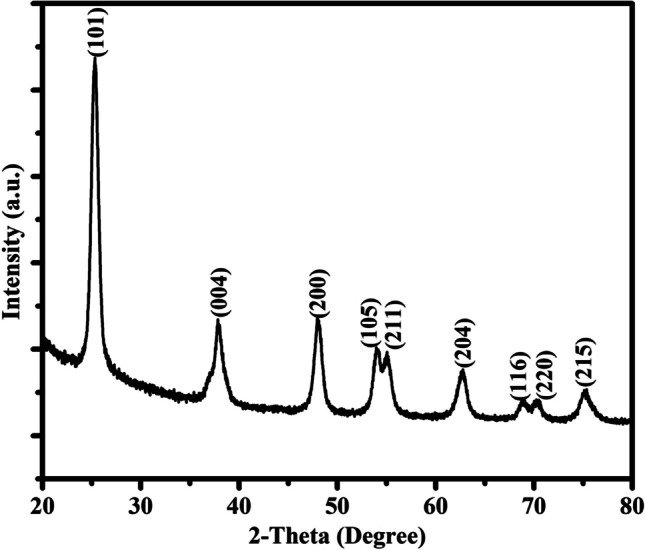
XRD pattern of TiO_2_-NPs

XRD pattern of the TiO_2_-NPs is shown in Fig. 1 and it indicates that the sample possesses an anatase phase and there are no peaks corresponding to the rutile phase. Intense reflections reveal the high crystallinity of the sample. The reflections at 2θ values of 25.28°, 37.85°, 48.04°, 54.17°, 55.13°, 62.68°, 68.98°, 70.18°, and 75.09°, correspond to (101), (004), (200), (105), (211), (204), (116), (220) and (215) crystal planes of the tetragonal phase (space group I41/amd) of TiO_2_ [37–39] and it matches well with the pattern of the database (PDF 00-004-0477). Debye–Scherrer equation (Eq. 1) is used to calculate the crystallite size of the TiO_2_-NPs.

$${\text{D}} = \frac{0.9\lambda }{{\beta \cos \theta }}$$

where λ represents the wavelength of the X-ray radiation (Cu Kα = 0.15418 nm), θ represents the Bragg diffraction angle, β represents the full width at half-maximum height, and D represents the average crystallite size. The obtained crystallite size for TiO_2_-NPs is 9.8 nm.

**Fig. 2:**
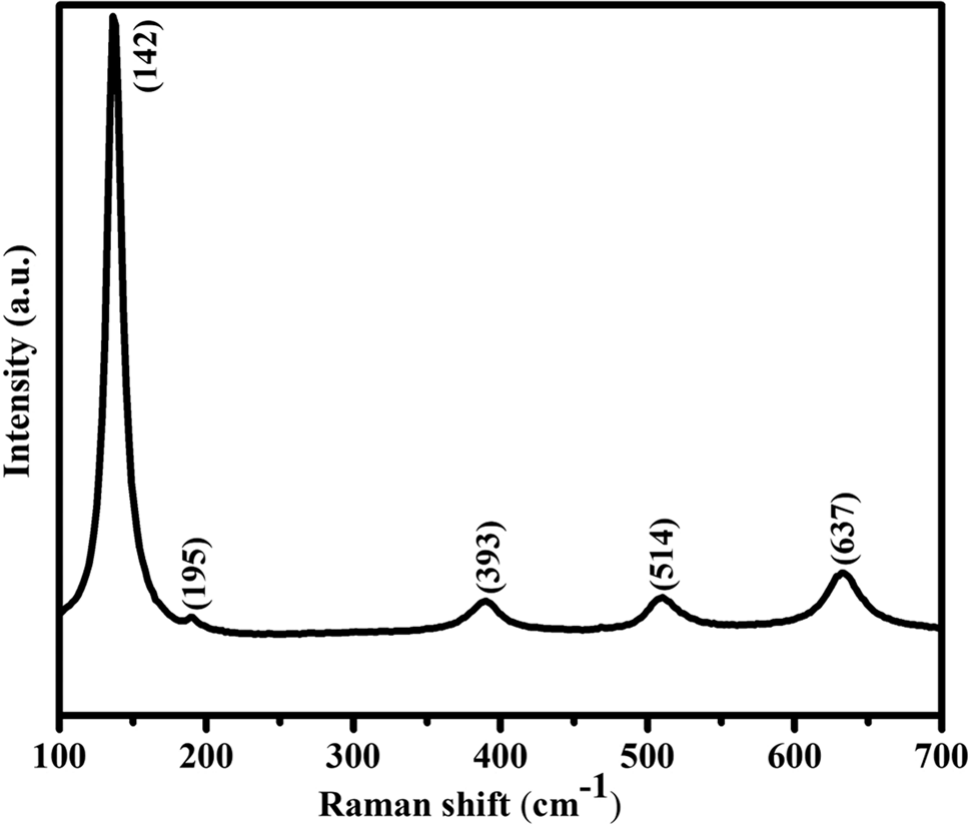
Raman spectrum of the anatase TiO_2_-NPs

The Raman spectrum was used to further confirm the phase and purity of the synthesized TiO_2_-NPs to provide a deeper understanding of the structure (Fig. 2). The trigonal anatase phase has a space group of I41/amd and its local symmetry is D2d [40]. The characteristic peaks centered at 142 (E_g_), 195 (E_g_), 393 (B_1g_), 514 (A_1g_), and 637 cm^−1^ (E_g_) demonstrate the anatase phase, without any band for rutile or brookite phase [41]. The band located at 637 cm^−1^ (E_g_) corresponds to the Ti–O stretching mode and the band that appeared at 397 cm^−1^ (B_1g_) refers to the O-Ti–O bending mode [42]. Thus, there is good agreement between the Raman spectrum and XRD data, confirming the existence of the high-purity TiO_2_ anatase phase.

**Fig. 3:**
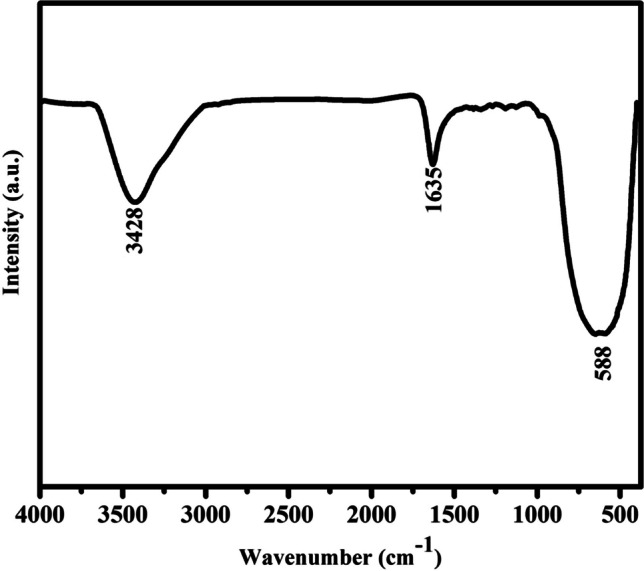
FTIR spectrum of the anatase TiO_2_-NPs

Figure 3 displays the FTIR spectrum of anatase TiO_2_-NPs. The broad band centered at 500–600 cm^−1^ corresponds to the Ti–O-Ti bending vibration in the TiO_2_ structure. The broad band centered at 3600–3400 cm^−1^ refers to the intermolecular interaction of the -OH groups in H_2_O molecule present on TiO_2_ surface. The band at 1635 cm^−1^ refers to signature -OH bending vibration [43].

**Fig. 4:**
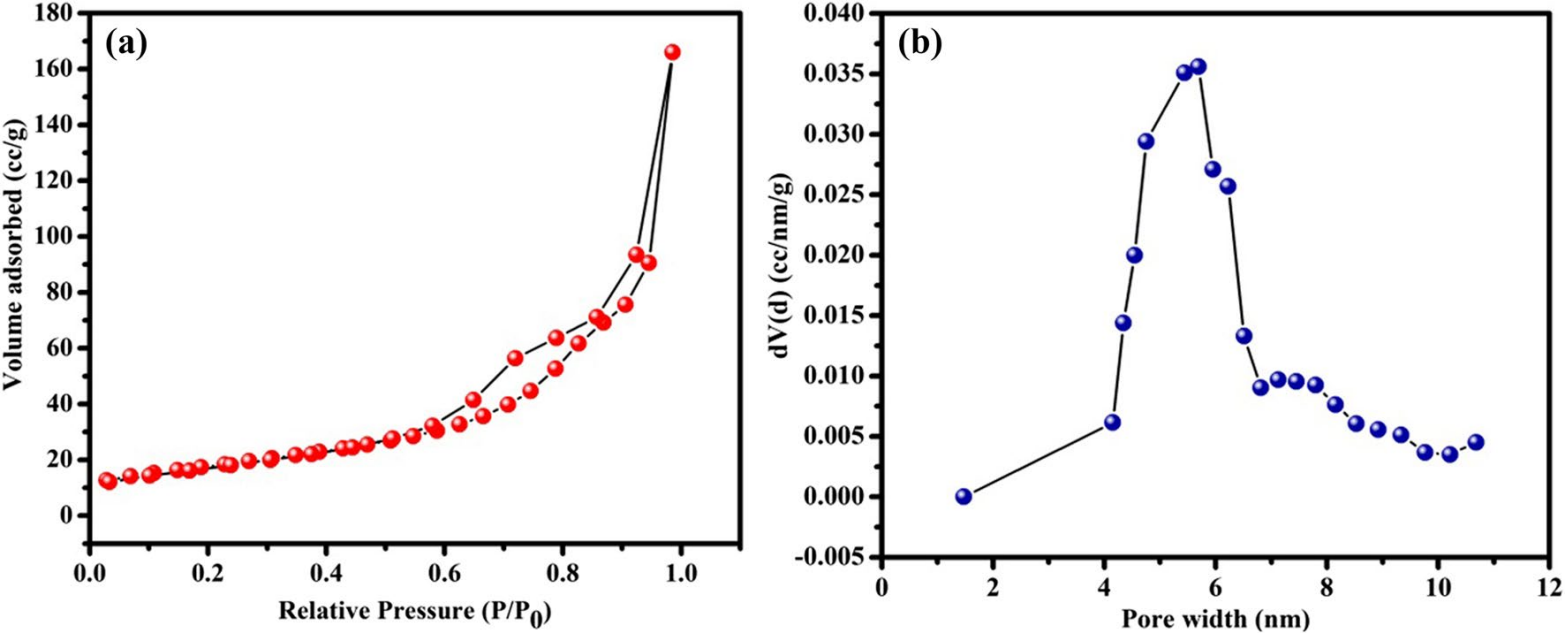
**a** N_2_ adsorption/desorption isotherms and **b** the pore size distribution of TiO_2_-NPs

Figure 4a represents the N_2_ adsorption–desorption isotherm of as-synthesized TiO_2_-NPs and is used to measure the surface area. The results revealed that at the relative pressure P/P_0_ of 0.99, the anatase TiO_2_-NPs possess type IV isotherm with a hysteresis loop. The mesoporous nature of the TiO_2_-NPs is suggested by the characteristic loop, and their presence can be attributed to the breakdown of biomolecules that have been capped on the surface of TiO_2_ and the release of CO_2_ during calcination. The adsorption isotherm curve is used to calculate the BET surface area of TiO_2_-NPs and the obtained value is around 63.2 m^2^g^−1^. The associated pore size distribution plot derived from the adsorption data using the Density Functional Theory (DFT) approach is shown in Fig. 4b. Results show that the sample has an average pore diameter of 5.43 nm and a pore volume of 0.257 cm^3^g^−1^.

**Fig. 5:**
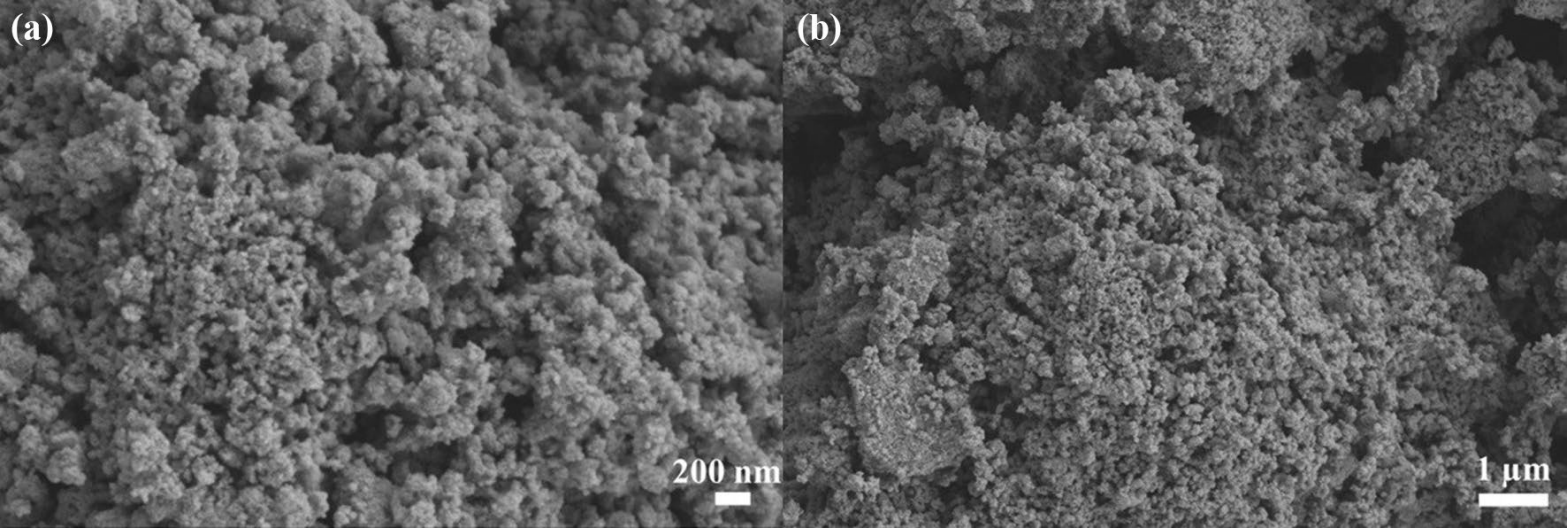
SEM images of TiO_2_-NPs at different magnifications, **a** at 25.0 kX and **b** at 10.0 kX

SEM analysis was used to investigate the surface morphology of TiO_2_-NPs, as shown in Fig. 5. SEM images indicate that the particles are agglomerated into a spherical sponge-like bunch of particles. The irregular primary particles form a loose and porous structure that makes it easy for lithium-ion diffusion and electrolyte infiltration into the bulk phase. Moreover, the smaller particles enable faster Li^+^ ion insertion and de-insertion in the TiO_2_ anode material, leading to improved charge and discharge process, especially at high C-rate cycling.

**Fig. 6:**
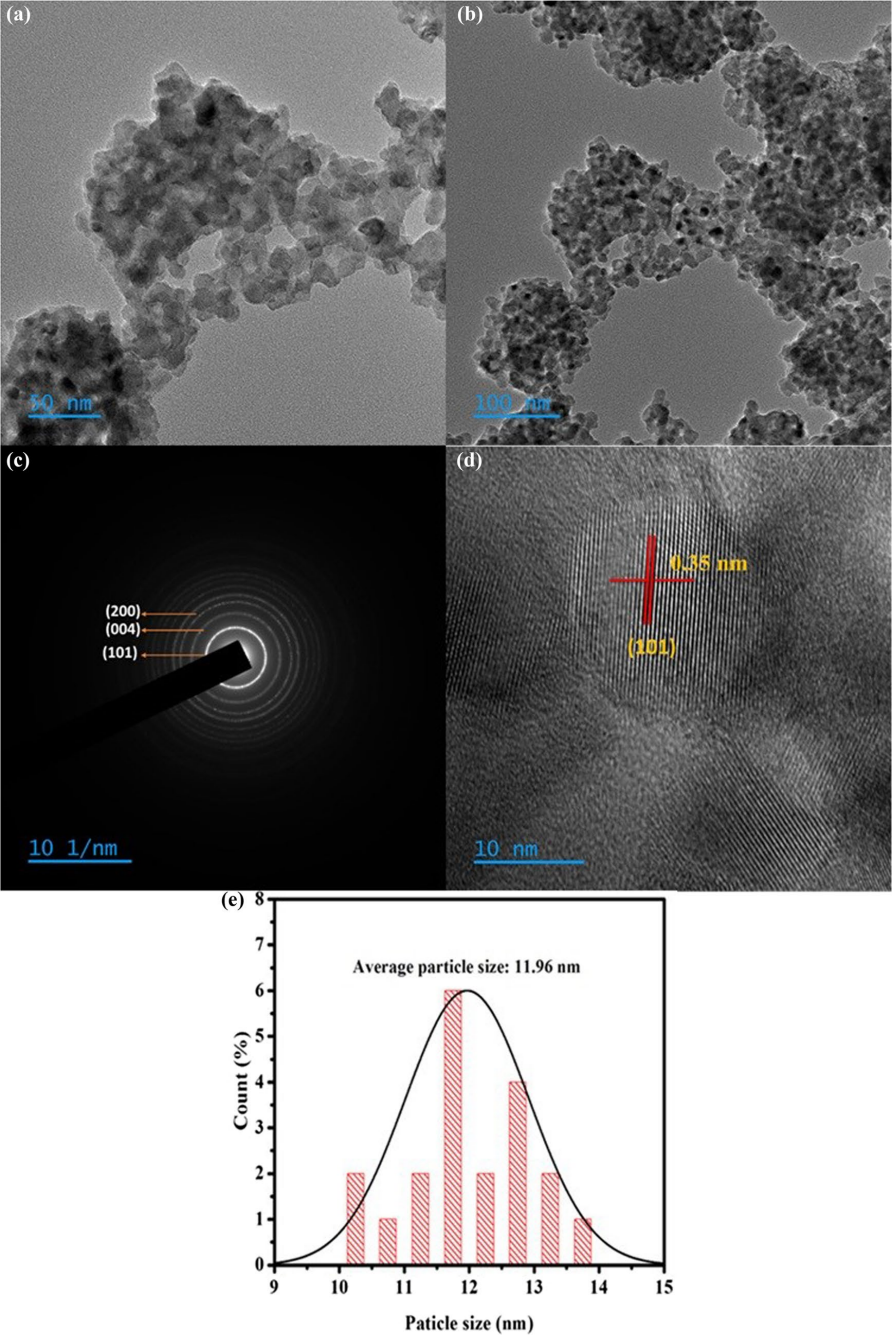
**a**,**b** TEM images of TiO_2_-NPs at different magnifications, **c** SAED pattern, **d** HR-TEM image of TiO_2_-NPs, and **e** the particle size distribution histogram of TiO_2_-NPs

TEM was employed to examine the actual shape and size of TiO_2_-NPs. The TEM image indicates that the synthesized TiO_2_-NPs are spherical in shape. The TEM image further demonstrates that the biomolecules in the beta extract act as capping agents, preventing the severe aggregation of TiO_2_-NPs. (Figs. 6a and b). The average particle size of each grain is 12 nm, which is comparable with the estimated crystallite size from XRD. The diffused rings in the selected area electron diffraction (SAED) pattern (Fig. 6c) indicate the polycrystalline nature of the synthesized TiO_2_-NPs. Moreover, it confirms the findings from XRD and Raman studies that the final TiO_2_ possesses an anatase phase. Furthermore, the SAED pattern strongly supports the XRD findings by showing the growth of nanoparticles along the (101), (004), and (200) planes. The HR-TEM images shown in Fig. 6d show lattice fringes with a d-spacing of 0.35 nm, corresponding to the (101) plane of highly crystalline tetragonal anatase TiO_2_. The Fig. 6e represents the particle size histogram of TiO_2_-NPs, which indicates that the average particle size is ~ 12 nm.

### Electrochemical performance of TiO_2_-NPs as anode material

**Fig. 7:**
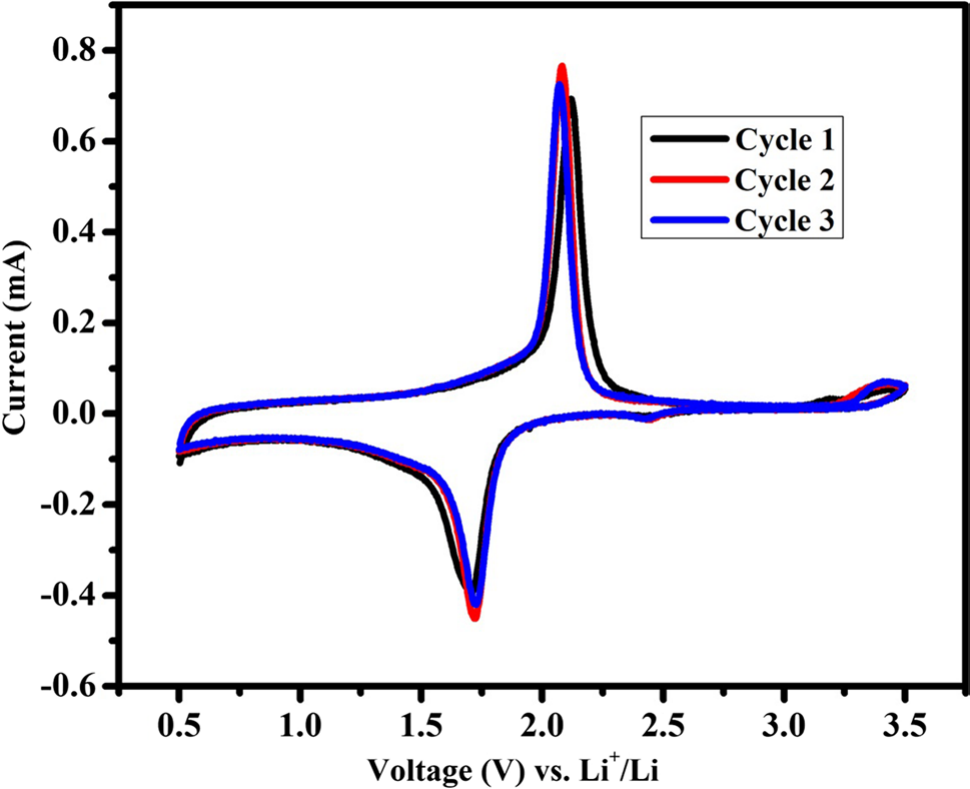
CV plots of TiO_2_-NPs in the voltage window of 0.5–3.5 V at a scan rate of 100 µV s^−1^

The electrochemical performance of TiO_2_-NPs was studied in CR2032 coin cells with Li metal as the counter electrode. At a scan rate of 100 µV s^−1^, half-cells were first subjected to CV measurements in the voltage window of 0.5 to 3.5 V vs Li^+^/Li. Figure 7 shows the CV test results for the initial three cycles. To facilitate lithium-ion insertion into the TiO_2_ crystal structure, the half-cell was initially discharged. Thus, a change in the valence state of titanium from Ti^4+^ to Ti^3+^ takes place. The Li-ion insertion (reduction peak) and extraction (oxidation peak), which occurred during the cathodic and anodic sweeps, respectively, are illustrated by the strong peaks at 1.72 and 2.12 V. In crystalline anatase electrodes, the separation of cathodic and anodic peaks is prevalent. These peaks show the exceptional reversibility of the anatase TiO_2_-NPs as an insertion host because during discharging Ti^4+^ was converted to Ti^3+^ and then oxidized to Ti^+4^ during charging. Sharp cathodic/anodic peaks during electrochemical Li^+^ intercalation/de-intercalation demonstrate the two-phase reaction mechanism in accordance with the following reaction: TiO_2_ + xLi^+^  + xe^−^ ↔ Li_x_TiO_2_ [44]. Nevertheless, a slight deviation in the peak position is seen in the succeeding cycles which may be due to the small stress developed in the TiO_2_ lattice during Li^+^ insertion/extraction process.

**Fig. 8:**
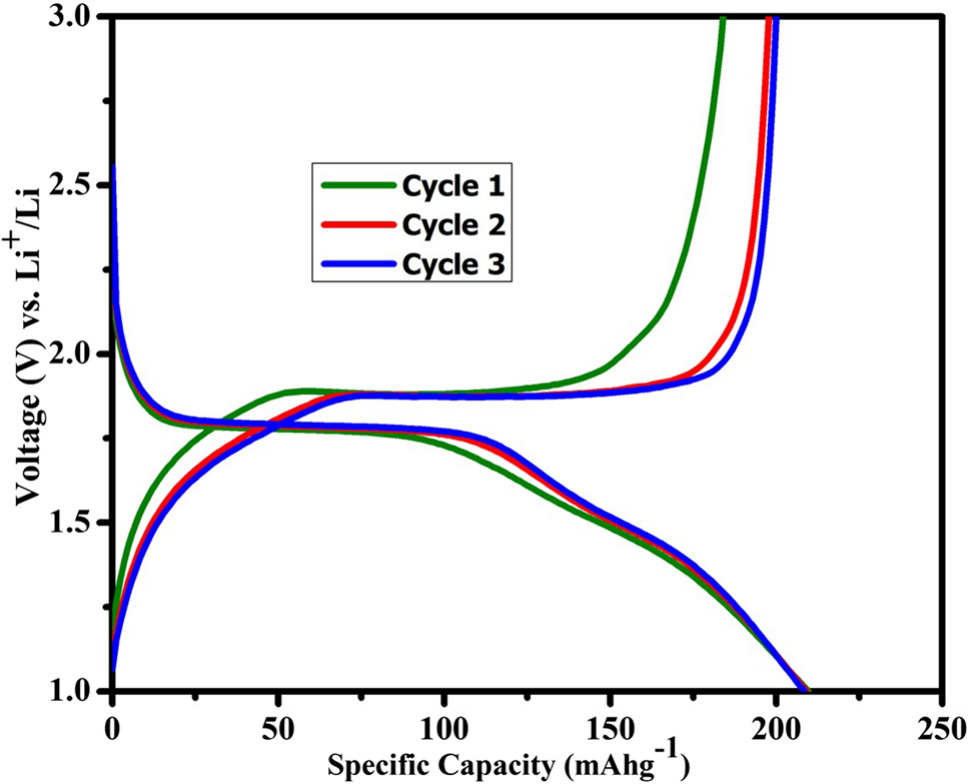
The first three charge–discharge curves of anatase-TiO_2_-NPs cycled at C/10 rate in the voltage window 1.0–3.0 V

The galvanostatic charge–discharge curves of TiO_2_-NPs in the voltage window of 1.0–3.0 V (C/10 rate) are shown in Fig. 8. In the first step, the half-cell was discharged to intercalate Li^+^ ions into the TiO_2_ lattice. Due to the loss of symmetry in the y direction during the lithium-ion intercalation, the unit cell symmetry of anatase undergoes a first-order phase transition from its initial tetragonal (I41/amd) structure to the orthorhombic (Pmn21) structure [30]. This phase transition happens simultaneously with a spontaneous phase separation of the lithium-poor (Li_0.01_TiO_2_) phase into the lithium-rich (Li_0.69_TiO_2_) phase, which was previously observed by several investigations [45, 46]. Though anatase TiO_2_-NPs have a theoretical specific capacity of 335 mAh g^−1^, the actual capacity that may be achieved is much lower due to the significant Li–Li repulsion in the Li_x_TiO_2_ framework at a higher degree of insertion, i.e., x > 0.5. In the present work, the anatase TiO_2_-NPs delivered an initial discharge capacity of 209.7 mAh g^−1^, which is equivalent to inserting 0.62 mol of lithium per formula unit. During the initial charging, the half-cell exhibited a capacity of 184 mAh g^−1^ (0.55 mol lithium per formula unit). The irreversible capacity during the initial discharge–charge process is ~ 25.7 mAh g^−1^ (corresponds to 0.076 mol Li), which is lower than other previously published works (Table 1). The charge–discharge curves show shortened plateaus at 1.78 V and 1.9 V. The plateau at 1.78 V refers to the Li-ion insertion (discharge) and the plateau at ~ 1.9 V refers to the Li-ion de-insertion (charge) [47]. The existence of a plateau region in the discharge–charge process demonstrates that the Li insertion occurs via a two-phase reaction mechanism and is in good agreement with CV peaks.

**Table 1:** A comparison of electrochemical performances of anatase TiO_2_ samples prepared in this work with previously reported materials

Material	Potential window (V)	Initial discharge capacity in mAhg^−1^	Irreversible capacity during first discharge- charge process (mAhg^−1^)	Cycle life, Capacity in mAhg^−1^ (Rate and no of cycles is given in parenthesis)	Rate capability (Rate is given in parenthesis)	Reference
Bio-TiO_2_	1.0–3.0	230	24	164 (33 mAg^−1^, 60)	46 (2 Ag^−1^)	Kashale et al. [51]
Anatase TiO_2_	1.0–3.0	221	32	182 (C/10, 30)	45 (30C)	El-Deen et al. [52]
Rutile and Anatase Composite TiO_2_ Nanotube (450 °C)	1.0–3.0	180	7	160 (C/10, 100)	62 (3C)	Wei et al. [53]
Rutile and Anatase Composite TiO_2_ Nanotube (600 °C)	1.0–3.0	230	20	185 (C/10, 100)	60 (3C)	Wei et al. [53]
TiO_2_ (B) nanosheets	1.0–3.0	245	84	162 (C/10, 25)	–	Wu et al. [54]
TiO_2_-nanobelt	1.0–3.0	250	–	110 (C/10, 10)	–	Luo et al. [55]
TiO_2_-230 °C	1.0–3.0	203.3	62.5	100 (0.12C, 10)	–	Luo et al. [56]
Lotus-root shaped TiO_2_	1.0–3.0	120	–	89 (0.5C, 100)	67 (2C)	Choi et al. [57]
Anatase TiO_2_ nanotubes	1.0–3.0	123	–	142 (36 mA g^−1^, 100)	122 (144 mA g^−1^)	Fasakin et al. [58]
Porous anatase TiO_2_-NPs	1.0–3.0	225	52	183 (5C, 20)	161 (10C)	Li et al. [59]
Anatase TiO_2_-NPs	1.0–3.0	209.7	25.7	148.7 (5C, 100)	149.5 (20 C)	This work

**Fig. 9:**
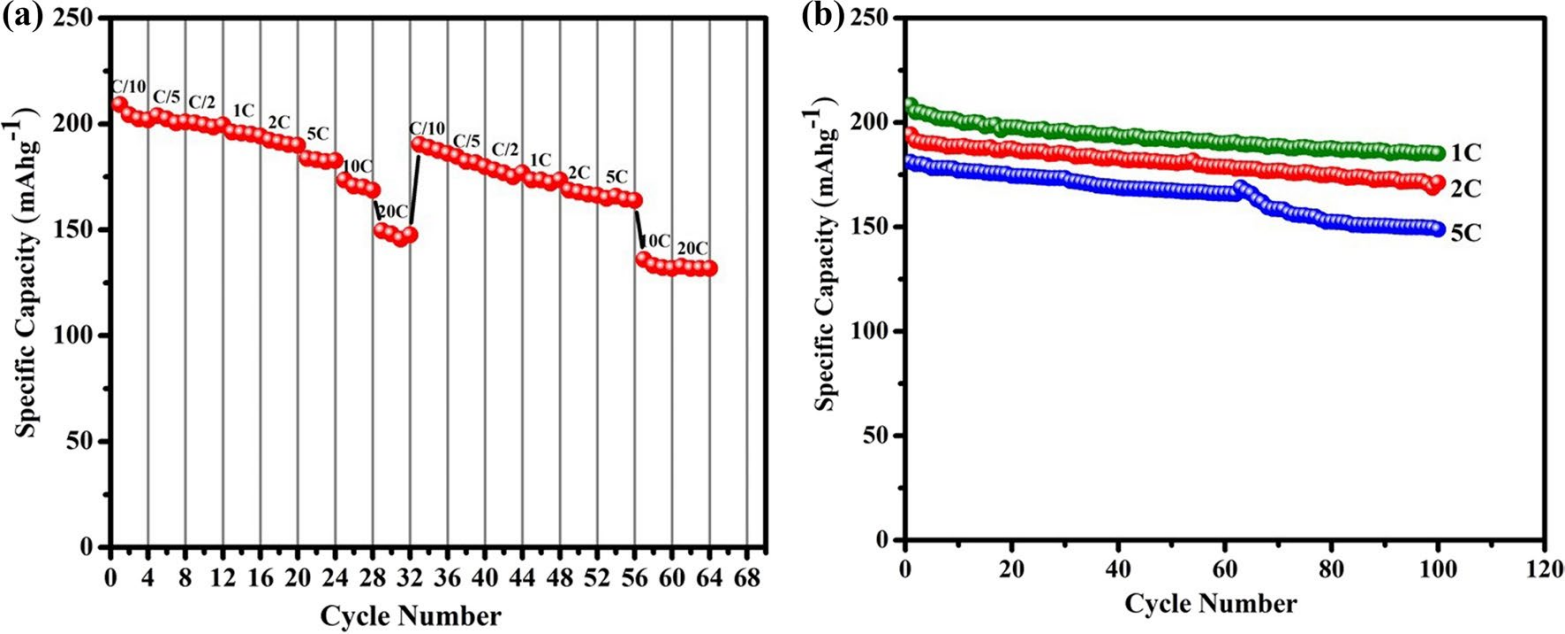
**a** Rate capability, and **b** cyclability profiles of the Li/TiO_2_ half-cell

**Fig. 10:**
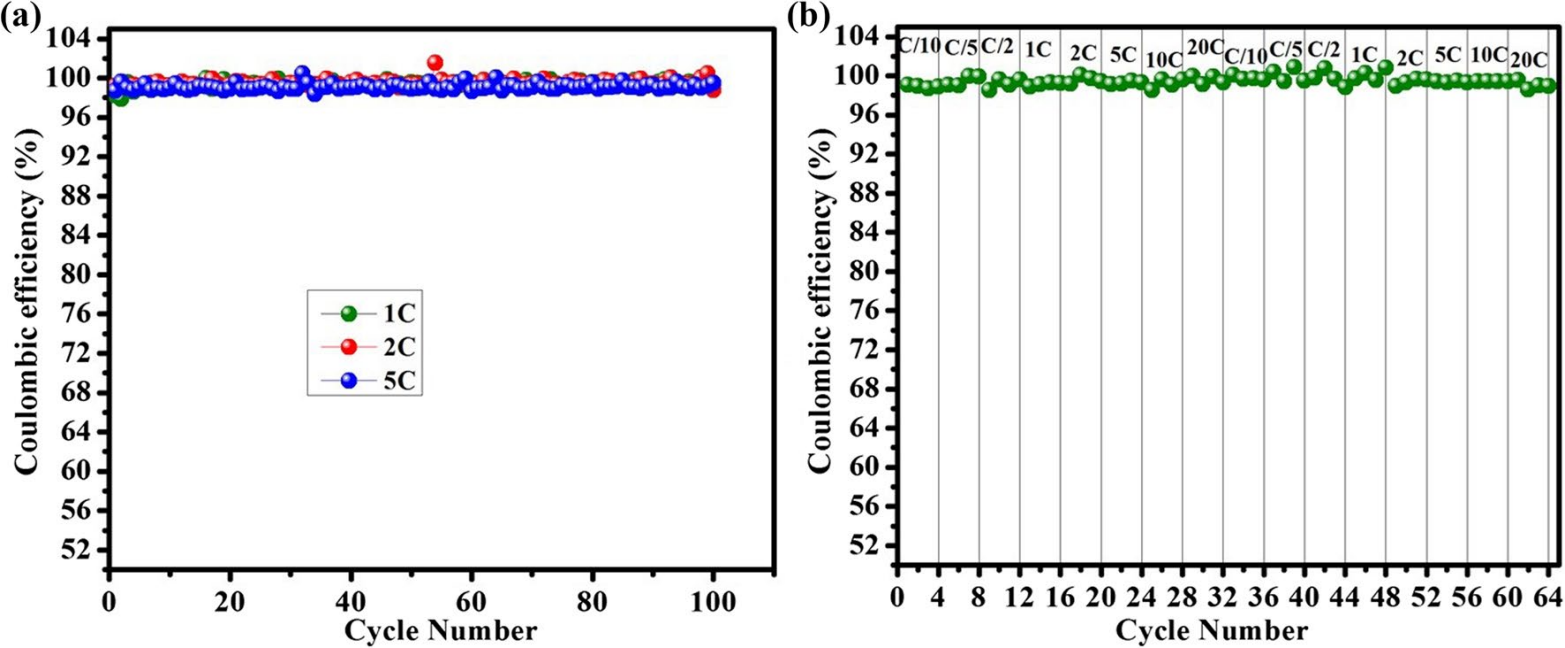
Coulombic efficiency of Li/TiO_2_ half-cell during **a** cycling, and **b** rate performance

The cycling performance and rate capability of anatase TiO_2_-NPs are shown in Fig. 9. The rate capability study is employed to examine the stability and versatility of the Li/TiO_2_ half-cell. Figure 9a shows the rate performance of Li/TiO_2_ half-cells at different current rates. The cell delivered discharge capacities of about 209.7, 203, 200, 196, 192, 183, 173, and 149 mAh g^−1^ at various current densities of C/10, C/5, C/2, 1C, 2C, 5C, 10C, and 20C, respectively. Figure 9a indicates that as the rate capability tests are repeated and the electrode cycled back at a high C-rate (20C), the material still delivers a discharge capacity of 132 mAhg^−1^. Thus, this gives evidence of the structural stability and electrochemical reversibility of the TiO_2_-NPs sample. Figure 9b demonstrates the cyclability of Li/TiO_2_ half-cell at three different current rates (1C, 2C, and 5C) for 100 cycles. The results revealed that the cell exhibited capacity retentions of 88.9% at 1C, 88.1% at 2C, and 81.2% at 5C rate at the end of 100 cycles. Figure 10 represents the coulombic efficiency of Li/TiO_2_ half-cell during cycling, and rate performances and the results indicate that during both studies the coulombic efficiency is ~ 100%. As compared with previously reported work, the present bio-mediated anatase TiO_2_-NPs exhibited better cycling and rate performances (Table 1).

**Fig. 11:**
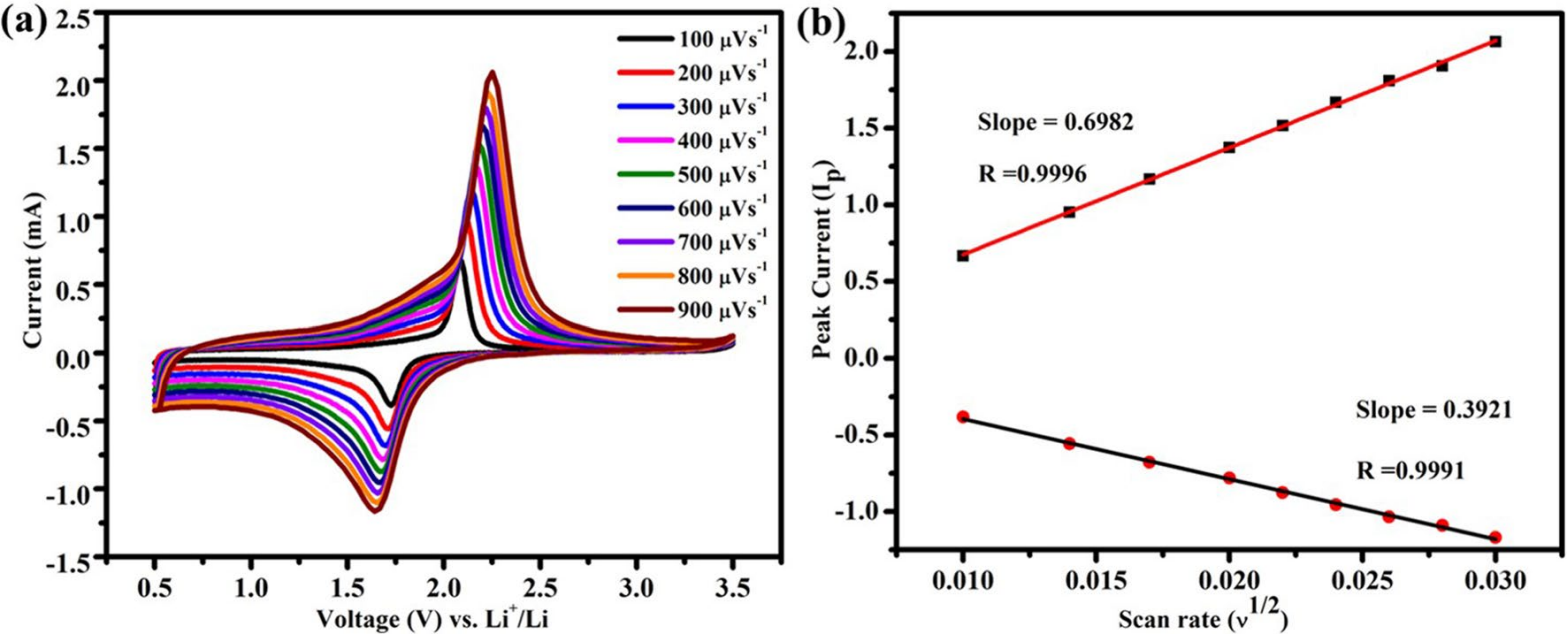
**a** CV curves of Li/TiO_2_ half-cell at different scan rates (from 100 µVs^−1^ to 900 µVs^−1^) and, **b** the plot of υ^1/2^ vs. I_p_

Cyclic voltammetry at various scan rates (100–900 µV s^−1^) was used to investigate the lithium-ion diffusion coefficient, D_Li_ in TiO_2_-NPs (Fig. 11a). As the scan rate increases, the reduction peaks shift toward lower voltage and the oxidation peaks gradually shift toward high voltage. Figure 11b depicts the relationship between the square root of the scan rate (ν^1/2^) and peak current (I_p_). The diffusion coefficient was calculated by Randles Sevcik equation [48–50],

$${\text{I}}_{{\text{p}}} = 2.69 \times 10^{5} {\text{n}}^{3/2} {\text{AD}}^{1/2} C_{{{\text{Li}}}} v^{1/2}$$

where n is the number of electrons transferred, A is the surface area of the electrode, D is the lithium-ion diffusion coefficient, C_Li_ is the concentration of Li^+^ ion in the electrode, ν is the scan rate and I_p_ is the peak current. The diffusion coefficient of Li^+^-ions calculated based on both anodic and cathodic peaks for the TiO_2_-NPs is 5.72 × 10^–11^ cm^2^s^−1^ and 1.86 × 10^–10^ cm^2^s^−1^ for de-lithiation and lithiation processes, respectively. Thus, the higher diffusion coefficient is responsible for the superior rate and cycling performance of the present anatase TiO_2_-NPs.

## Conclusions

In summary, anatase TiO_2_-NPs were successfully synthesized through a green-mediated process using Beta vulgaris (Beetroot) extract. The material was characterized by X-ray diffraction (XRD), Raman spectroscopy, infra-red (IR) spectroscopy, scanning electron microscopy (SEM), transmission electron microscopy (TEM) analysis, and evaluated as anode in lithium-ion cells. Furthermore, an aqueous binder (a combination of CMC and SBR) is employed for electrode processing. The electrochemical performance of the Li/TiO_2_ half-cell was evaluated in the potential window of 1–3 V at C/10 rate. The cycling stability and rate capability studies demonstrate that the material exhibits superior performances than the previously reported TiO_2_-NPs material. Even at 20 C rate, the material delivered a discharge capacity of 149 mAhg^−1^. Hence, the present work highlights a completely greener approach for both material synthesis and electrode processing. This work will be highly beneficial for producing anodes for high-power LIBs in a cost-effective and environmentally friendly route.

## Data Availability

All data generated or analyzed during this study are included in this published article.
